# Molecular Complexity Calculated by Fractal Dimension

**DOI:** 10.1038/s41598-018-37253-8

**Published:** 2019-01-30

**Authors:** Modest von Korff, Thomas Sander

**Affiliations:** Scientific Computing Drug Discovery, Idorsia Pharmaceuticals Ltd., Hegenheimermattweg 91, CH-4123 Allschwil, Switzerland

## Abstract

Molecular complexity is an important characteristic of organic molecules for drug discovery. How to calculate molecular complexity has been discussed in the scientific literature for decades. It was known from early on that the numbers of substructures that can be cut out of a molecular graph are of importance for this task. However, it was never realized that the cut-out substructures show self-similarity to the parent structures. A successive removal of one bond and one atom returns a series of fragments with decreasing size. Such a series shows self-similarity similar to fractal objects. Here we used the number of distinct fragments to calculate the fractal dimension of the molecule. The fractal dimension of a molecule is a new matter constant that incorporates all features that are currently known to be important for describing molecular complexity. Furthermore, this is the first work that reveals the fractal nature of organic molecules.

## Introduction

### Background of molecular complexity

Molecular complexity has been a topic of research for more than sixty years. Still, no common definition of the term ‘molecular complexity’ is currently available, even though it is a matter of daily discussions in synthetic organic chemistry laboratories. The lack of a common definition for molecular complexity recently resulted in a crowd-sourced approach^1^. A lot of the work mentioned below was triggered by the need to assess molecular complexity related to chemical synthesis. Molecular complexity is an intrinsic feature that depends only on the molecular structure but, nevertheless, provides an estimator for the synthetic effort^2^. Conversely, the accessibility of synthesis is a combination of intrinsic and extrinsic molecular properties as it depends on the applied educts, reagents and reaction conditions.

As pointed out above, molecular complexity naturally is a topic for chemistry; however, it originates in biology. In 1955, Rashevsky^3^ aimed to calculate the information content of a living organism and, therefore, needed to know the information content of its integral parts such as organic molecules. Rashevsky suggested a formula to calculate information content of molecules, based on the Shannon entropy^4^. Rashevsky used terms ‘complexity’ and ‘information content’ synonymously. Decomposing a graph into its sub-graphs, while considering symmetry, was used by Mowshowitz for measuring graph complexity^5^. Based on the work of Rashevsky, Bonchev used the topological information content to calculate molecular graph features^6^. The topological complexity was based on the total number of connected subgraphs of the molecule^7^. As many other complexity measures, the topological complexity increases with the number of atoms and bonds. Independently from Rashevsky and Bonchev’s work, Bertz published a method to calculate a complexity index for a molecule^8^, also relying on the Shannon entropy. Bertz introduced the idea of using graph theoretical invariants for complexity calculations on chemical structures. A central term in his complexity considerations was the number of ways a subgraph can be cut out of a graph. In a more recent publication, Bertz and Herndon took the total number of subgraphs as measure for molecular complexity^9^. Hendrickson *et al*.^10^ later simplified the algorithm of Bertz and introduced correction for molecular symmetry. Randic introduced the ‘molecular connectivity index’ which he employed to calculate molecular complexity^11^. Based on the ideas of Bertz, Proudfoot has recently derived a molecular complexity measure that summed up the complexity of every atom environment in a molecule^12^.

The domain-centered approach and the limited set of chemical elements and substructures used in organic chemistry, especially in medicinal chemistry, triggered the idea to develop another class of complexity measures for organic molecules by counting molecular features. Whitlock used the number of rings, unsaturated bonds, hetero atoms, and chiral centers to calculate the complexity of natural products^13^, an algorithm later simplified by Borone and Chanon^14^. Similarly, Allu and Oprea^15^ used the number of chiral centers, added fused rings, functional groups, and electronegativity to calculate molecular complexity. A more general approach was developed by Rücker and Rücker^16^, utilizing a program to count walks on a molecular graph. Their work is also an example for a fluent transition between graph theoretical approaches and molecular feature based approaches.

Besides the efforts to describe molecular complexity with a single value, physico-chemical descriptors have been used for decades to obtain a number for molecular features like complexity^17,18^. It was shown by Sheridan *et al*. that some combinations of physico-chemical descriptors, such as the number of chiral centers, number of unique topological torsions, and the number of unique atom pair descriptors, correlated with the perception of molecular complexity by medicinal chemists^1^.

### From the Shannon entropy to the fractal dimension of molecules

Having reviewed the available literature on molecular complexity, we identified two major approaches used to calculate molecular complexity.

The first, graph theoretical approach evaluates substructure-related features and sums them up with the Shannon entropy formula that was initially developed to measure the content of binary information (1). For molecular complexity calculations, Rashevsky suggested using Shannon’s formula with *P*(*x*_*i*_) being the number of graph features for an atom or a substructure *x*_*i*_. This reflects the increase in both entropy and complexity of the molecule with increasing number of information fragments. However, increasing the size of a molecule does not necessarily correlate with its increasing molecular complexity in the eyes of chemists.

1 $$H(X)=-\,\sum _{i=1}^{n}\,P({x}_{i})\,lo{g}_{2}P({x}_{i})$$

The other approach uses weighting schemes to combine molecular features into a complexity number. A disadvantage of using weighting schemes is that it needs parameterization that can only be done with a limited number of molecules. A complexity calculation with a weighting scheme will only be valid for the classes of molecules the parameterization was done for.

Intuitively, organic chemists agreed that certain features that bring variety into the molecular graph (e.g., chiral centers, sp3 carbons, fused rings, bridged rings, and hetero atoms) also increase molecular complexity but only if these features are not repetitive. On the other hand, repetition of identical or very similar molecular features lowers the complexity of a molecule. Based on this point, we set out to calculate molecular complexity in a completely new way.

**Figure 1:**
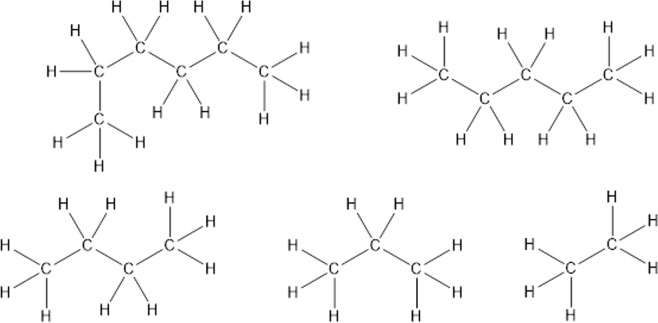
n-Hexane and four distinct alkanes obtained by successively removing one bond and one atom, followed by saturation with hydrogen.

During our work on repetitive molecular features we realized that every molecular substructure displays self-similarity to its parent structure. Self-similarity means that an object is similar to a part of itself. Let us derive the concept of self-similarity for organic molecules at the example of n-hexane, a linear alkane (Fig. 1). A linear alkane consists of a chain of carbon atoms saturated with hydrogen atoms. Removing one of the two outmost carbon-atoms and the connecting bond creates a new chain with one bond less. To the chain carbon-atom from where the bond was removed, a hydrogen atom has to be added for completing the saturation of the chain. Resulting is a new alkane, pentane, that is slightly smaller than the start alkane but very similar to it. If the other outmost bond is removed from the hexane carbon chain, again, a chain with five carbon atoms is obtained. This fragment can be transferred into pentane by saturation with hydrogen. So, independently which outermost bond was removed the same fragment is obtained. These two fragments are non-distinct or isomorphic. The successive bond removal can be repeated up to the last bond. The resulting alkane will always be similar to the parent chain.

**Figure 2:**
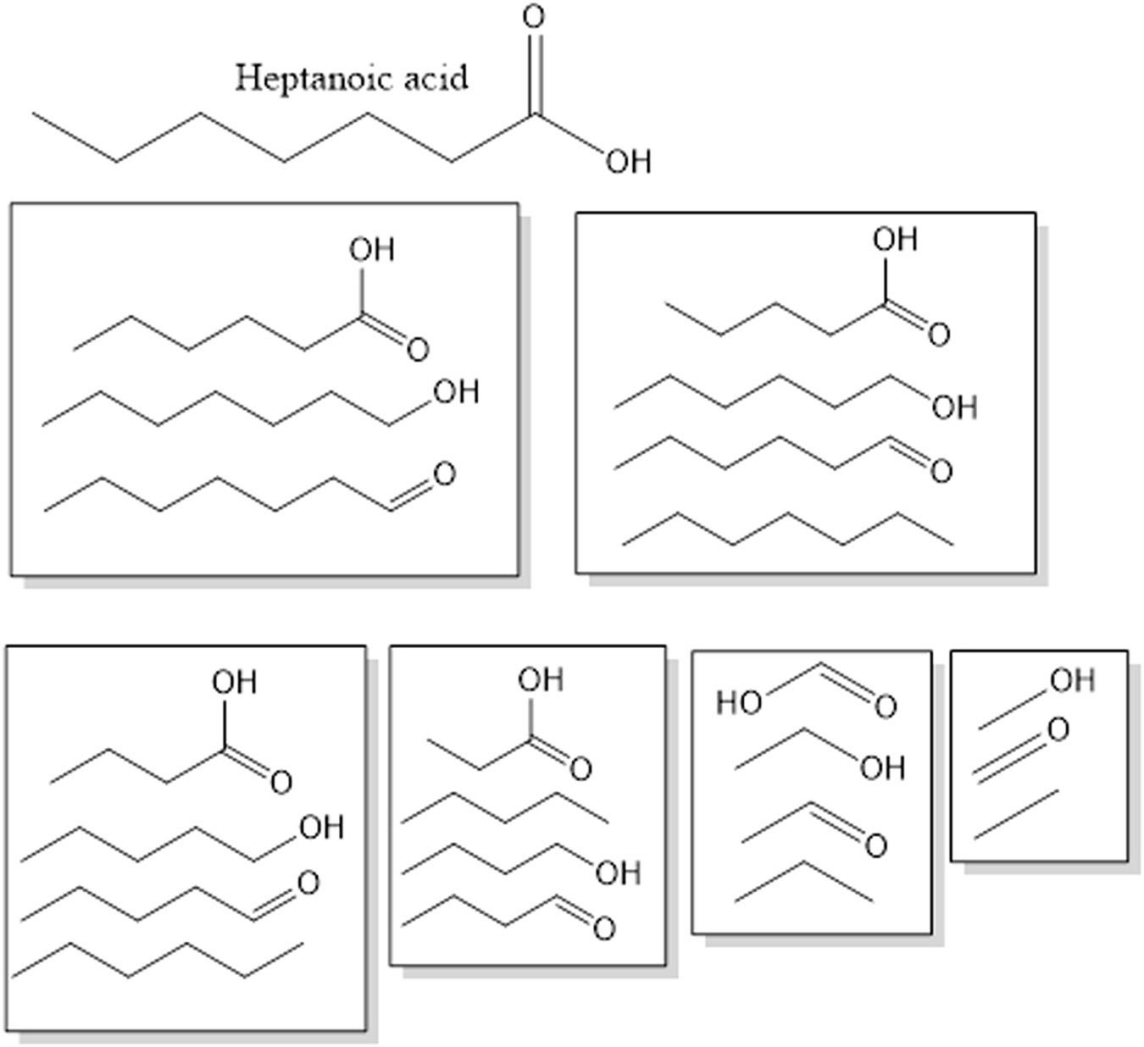
Heptanoic acid and its distinct substructures. Grouped by bond counts.

A bit more structural variety is shown by heptanoic acid (Fig. 2). For this and all the following molecules we were using the bond line notation, where vertices and the ends of each line represent carbon atoms and hydrogen atoms attached to them are not shown. In bond line notation, heptanoic acid consists of nine atoms and eight bonds. Removing one bond and one atom on either end of the molecule results in three distinct substructures with eight atoms and seven bonds. Repeating this procedure with these three substructures yields four new substructures with seven atoms and six bonds. The removal of one atom and the adjacent bond can be continued until only fragments with one bond and two atoms remain. For heptanoic acid exists three distinct fragments with one bond and two atoms. Every fragment with any bond count is similar to its parent molecule.

**Figure 3:**
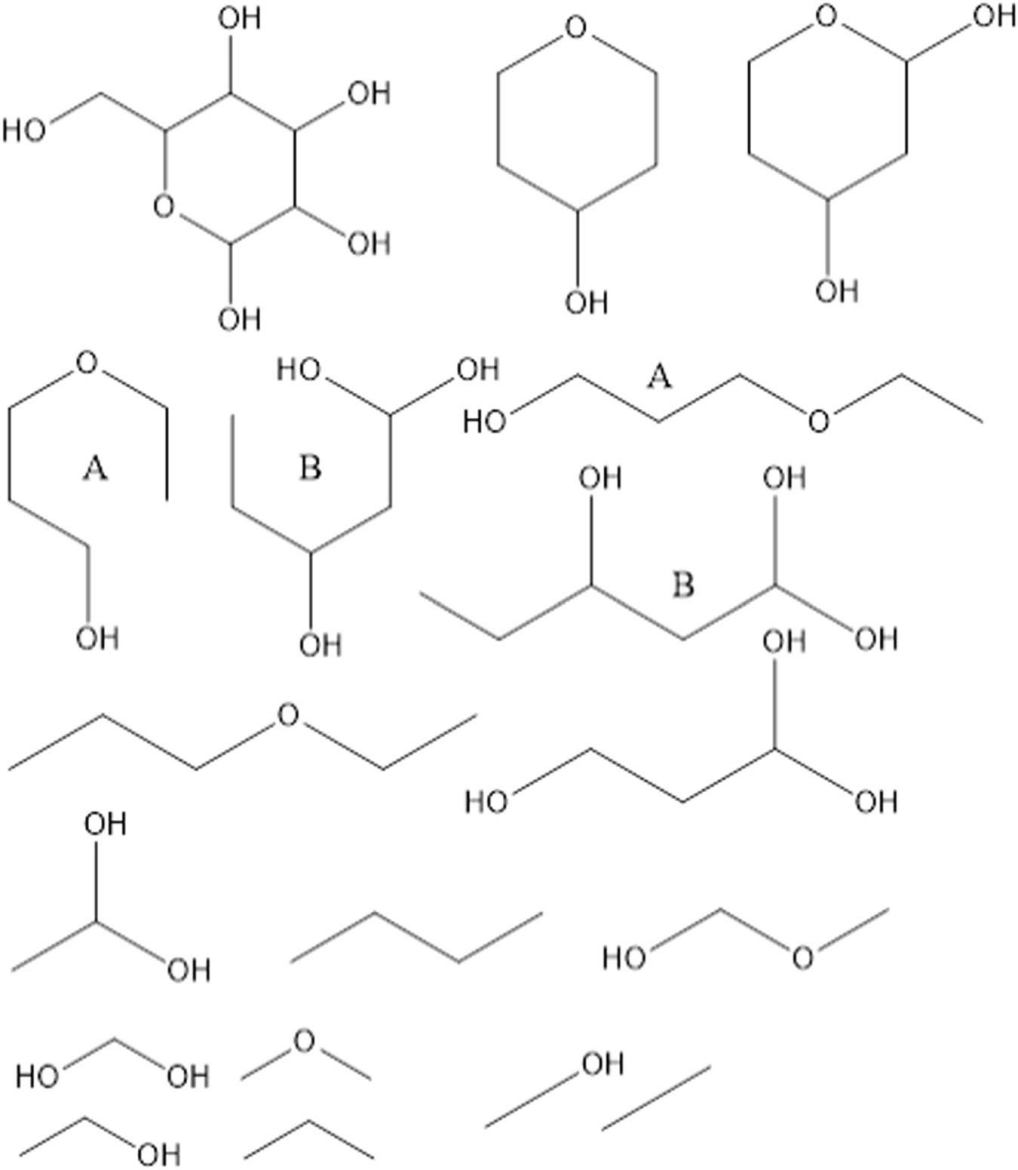
Glucose and some of its distinct subgraphs. Subgraphs A and B are shown twice in both bent and elongated forms.

Glucose is an important biomolecule. Figure 3 shows glucose and some of its smaller substructures. Two substructures (A and B) are shown in both bent and elongated forms. These subgraphs were created from parent ring fragments by removing a bond without removing an atom.

The obvious similarity of molecular subgraphs to their parent graphs triggered the idea to measure the self-similarity. Self-similarity is a well-established concept in mathematical and physical sciences. Measuring self-similarity started with Hausdorf^19^ and was established by Mandelbrot^20^ with the calculation of fractal dimensions. According to Mandelbrot, a fractal dimension is a ratio providing an index of complexity by comparing how a detail of a pattern changes with the scale at which it is measured^21^. An index of complexity is exactly what we desired to measure. From the manifold of existing algorithms to determine fractal dimensions we decided to calculate the fractal dimension of molecules analogously to the widely used Minkowski-Bouligand dimension (2)^22^.

2 $$\dim (S)=\mathop{\mathrm{lim}}\limits_{\varepsilon \to 0}\frac{\mathrm{log}\,N(\varepsilon )}{\mathrm{log}\,(1/\varepsilon )}$$

**Figure 4:**
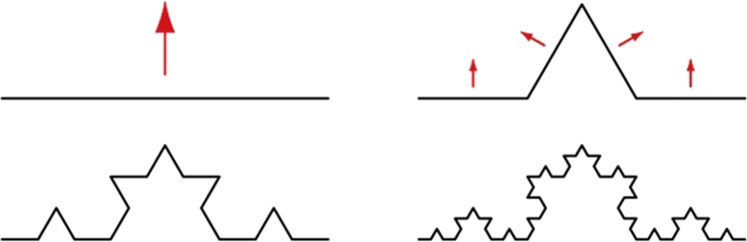
Koch curve.

With N as the number of objects and ε is the scale the objects were measured with. In practice, the fractal dimension is calculated by the slope that is determined by a linear regression of log(1/*ε*) versus log *N*(*ε*) for all ε. A common method to determine the Minkowski-Bouligand dimension is the box counting algorithm for two dimensional shapes. In the box counting algorithm, N equals the number of boxes that are needed to cover the shape for which the fractal dimension is determined. The scale ε is the side length of a box. With this scale, N boxes are needed to cover the shape. With a decreasing scale, ε increases the number of boxes N(ε) that are needed to cover the shape, i.e., both the nominator and the denominator of equation (2) increase with increasing N. The classic example to determine the Minkowski-Bouligand dimension with the box counting algorithm is the calculation of the fractal dimension for the coastline of Britain by Mandelbrot^20^. This publication brought the idea of fractal dimensions to the broad scientific community. However, there are simpler structures that allow studying fractal dimensions. To explain the similarities between molecules and fractals we looked for a self-similar structure that can be constructed based on a set of rules. The von Koch curve^23^ starts with a line, Fig. 4. The middle part of the line is replaced by a triangle consisting of two lines of equal length that is also equal to the length of the replaced part. By comparing the von Koch curve with the line notation of alkanes it becomes obvious that it can also represent a linear alkane. In line notation, a vertex of an angle represents a carbon atom with attached hydrogen atoms and lines represent bonds between the carbon atoms. For an alkane depicted as a Koch curve, the fractal dimension equals log(4)/log(3) ≈ 1.262.

**Figure 5:**
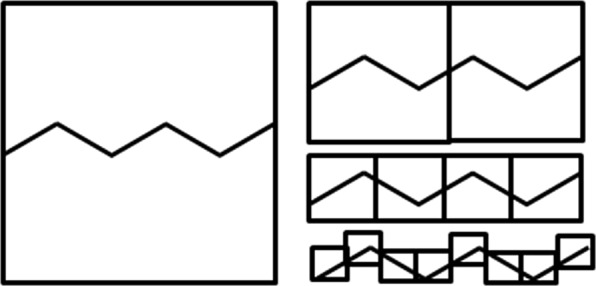
n-Hexane in line notation, covered by boxes for the box counting algorithm.

Although it is possible to represent an alkane with a von Koch curve, chemists prefer a simpler set of rules. The angle between two lines is set to 120 degrees. If the originating line is pointing upward, then the next line attached to it must point downwards, and vice versa. This results in a zigzag pattern, Fig. 5.

As this pattern is two-dimensional, the ‘box-counting’ algorithm can be applied. In this algorithm, the maximum side length ε_max_ of a square ‘box’ is set to one. The largest square covers the complete shape, representing the whole molecule. Consequently, the side lengths of smaller squares are less than one. We define the side length of the second square ε_2_ by ε_max_/2. All other square side lengths follow the same rule by dividing the side length of the previous square by 2. Table 1 shows the results, which are equal to 1 for all square side lengths, resulting is a slope of zero, which becomes the fractal dimension of the zig-zag pattern. Both the zig-zag pattern and the Koch curve are valid representations for alkanes. They differ in their fractal dimension, which makes the topological approach inappropriate for calculation of the fractal dimensions of molecules.

**Table 1:** Box counting algorithm applied to n-hexane in Fig. 5.

Side length square ε	Number of squares N	log(N)/log(1/ε)
1	1	Not defined
0.5	2	1
0.25	4	1
0.125	8	1

**Figure 6:**
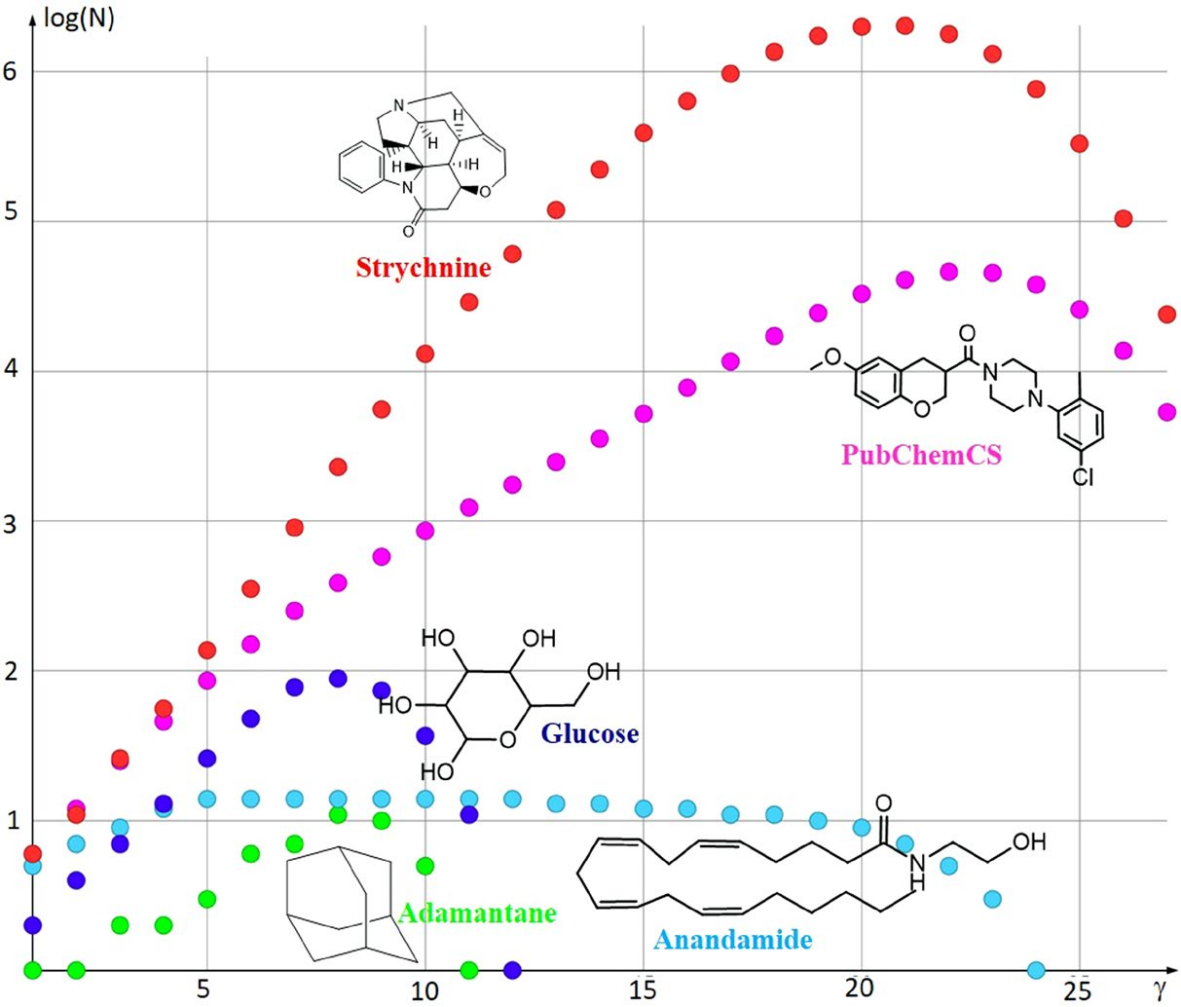
The number of distinct subgraphs (N) for five example organic molecules versus the bond count of the subgraphs (γ). The numbers of distinct subgraphs on the y-axis are given in logarithmic scale.

In any case, a topological representation of molecules is a very rough approximation of their three-dimensional structure. In line with this, the topological fractal dimension calculation is only possible for non-bridged alkanes, the simplest organic molecules. If a heteroatom is added, such as nitrogen or oxygen, the box counting algorithm cannot be applied any more. The heteroatom adds a new dimension that cannot be covered by the two-dimensional shape. With the hetero atom, the shape becomes a graph. Analyzing a graph with topological methods like the box counting algorithm would create invalid results. Also, the similarity of the extracted substructures to the parent molecule is not captured by the box counting algorithm and, therefore, calculation of the fractal dimension of a graph requires a different approach. Fortunately, the definition of fractals by Mandelbrot covers much more than shape-based objects. As already mentioned, the definition of complexity by Mandelbrot requires only an index that measures how the detail of a pattern is changing with the scale on which the index is measured. As already shown in Figs 1 to 3, the substructures are details of the molecule pattern. The scale is defined by the number of bonds in the substructure. In a molecule, the level of detail is increasing with an increasing bond count γ in the substructures, as it is shown in Fig. 6 and discussed in the results section. The lowest level of detail in the substructures is bond count one. Accordingly, a bond count of one is equal to the largest side length in the box counting algorithm. And the bond count with the largest numbers of substructures represents the highest level of details. This bond count γ_max_ corresponds to the shortest side length in the box counting algorithm. In other words, the number of bond counts *γ* is inversely proportional to the change in the side lengths *ε* of the box counting algorithm *ε* is always less than one and is approaching zero, *ε* → 0, at the highest level of detail. The bond count γ is increasing until it reaches the highest level of details *γ* → *γ*_*max*_. To bring the highest level of details γ_max_ into the appropriate format for the fractal dimension calculation, equivalent to equation 2, the limit is replaced by 1/*γ* → 0 This change must be reflected in the denominator and, thus, equation 2 is transformed into equation 3:

3 $${\rm{\dim }}(M)=\mathop{\mathrm{lim}}\limits_{1/\gamma \to 0}\frac{\mathrm{log}\,N(\gamma )}{\mathrm{log}(\frac{1}{1/{\gamma }_{max}})}$$

Thus, for the fractal dimension of a molecule the denominator of equation 2 changes from $\mathrm{log}(1/\varepsilon )$ to log(1/(1/γ_max_)). While the nominator stays unchanged, the number of features N that was defined as the number of boxes is now defines as the number of distinct subgraphs. The denominator can be simplified without changing the result.

4 $${\rm{\dim }}(M)=\mathop{\mathrm{lim}}\limits_{1/\gamma \to 0}\frac{\mathrm{log}\,N(\gamma )}{\mathrm{log}\,\gamma }$$

Equation 4 has the same form as equation 2. Thus, the fractal dimension for a molecule is calculated analogously to the Minkowski-Bouligand dimension by calculating the slope of log *γ*_*max*_ versus log *γ*_*max*_, with *γ*_*max*_ the number of bonds where the maximum number of distinct subgraphs occurred.

## Results

Figure 6 shows the distribution of the number of distinct subgraphs (N) on a semi-logarithmic scale for five examples of organic molecules. For all molecules, N increased monotonously with the increasing number of bonds in the subgraph (γ) until it reached a maximum (N_max_). Moreover, glucose, the molecule from a commercial supplier with the PubChem Id 22578173 (PubChemCS) and strychnine displayed a strictly monotonic graph. The maximum, be it a single peak or a plateau in case of a molecule with repetitive structural elements (anandamide), was followed by a steep decline in N. This repeating pattern allowed calculation of the fractal dimension of each molecule using equation 4 (Table 2).

**Table 2:** The maximum number of distinct fragments (N_max_), the corresponding bond count (γ_max_), and the fractal dimension (dim(M)) of five example molecules.

Name	Structure	N_max_	γ_max_	dim(M)
Adamantane		11	8	1.2
Anandamide		14	5	1.6
Glucose		89	8	2.2
PubChemCS		45,973	22	3.5
Strychnine		2,022,462	21	4.8

Adamantane is a highly regular alkane molecule, but in contrary to linear alkanes, it contains branches and bridged rings. Because of the bridged rings, it is impossible to apply the box-counting algorithm. So, the fractal dimension was only calculated by the maximum number of distinct fragments that could be cut out of the molecular graph. For all bond counts the number of distinct fragments was determined. The fractal dimension was calculated from the highest number of distinct fragments *N*_max_ and the corresponding bond count *γ*_*max*_. Instead of one distinct fragment, like in hexane, eleven distinct fragments were counted at *γ*_*max*_ of eight bonds. Such low quantity of distinct fragments resulted in a small fractal dimension of 1.2.

Anandamide consists of many more atoms and shows higher structural diversity than adamantane. It has also more atoms and bonds than glucose, nevertheless is the fractal dimension of glucose considerably higher. This finding comes without surprise, the number of distinct fragments in glucose is much higher than the number of distinct fragments in anandamide.

The molecule PubChemCS produced about 46,000 distinct subgraphs, which is almost three orders of magnitude above the number of distinct fragments for glucose. Hence, the fractal dimension of 3.5 for the PubChemCS molecule is just about one dimension above. This difference for the fractal dimensions of Glucose and the PubChem molecule is explained by the different bond counts at their γ_max_ values.

Strychnine had the maximum of about two million distinct fragments at γ_max_ of 21 bonds. This resulted in the highest fractal dimension of 4.8 among all analyzed molecules in this study. The high fractal dimension of strychnine nicely reflects its synthetic complexity. The first total synthesis of strychnine by Woodward^24^ was awarded by the Nobel Prize in chemistry.

In contrast, the linear alkane molecule n-hexane from Fig. 1 contains only one distinct subgraph at any number of bonds, resulting in fractal dimension of zero. This result is true for all linear alkanes, which is in line with the consensus of organic chemists that linear alkanes are the most simple organic structures. In terms of fractal dimension, linear alkanes are equivalent to a point in geometry. This is also identical with the result of the box counting algorithm for n-hexane in Fig. 5. Although the box counting algorithm can be applied to alkanes, such topological approach is inapplicable to more complex molecules.

## Conclusions

Here, we demonstrated for the first time that organic molecules show fractal properties as they can be subdivided into a number of subgraphs which show similarity to their parent graph. Because the fractal dimension of a molecule is clearly defined by the maximum number of distinct subgraphs at a certain bond count, the definition is even more robust than the original Minkowski-Bouligand dimension. For the calculation of the Minkowski-Bouligand dimension with the box-counting algorithm a number of scales is needed. However, this number has to be determined somehow, which introduces an additional degree of freedom into the calculation. For the calculation of the fractal dimension of molecules given in equation 4, the maximum level of detail is defined by the bond count *γ*_*max*_ in equation 4. Apparently there is some similarity between the Shannon entropy (equation 1) and the fractal dimensions of molecules (equation 4). Both formulas calculate the logarithm from the number of features. The difference is that the Shannon entropy builds the sum of the features while the fractal dimension derives the slope from the logarithmic number of distinct subgraphs per logarithmic number of bonds. The division by the logarithm of the number of bonds diminishes the influence of molecule size. The fractal dimension of molecules is a straightforward definition for molecular complexity. In contrast to Shannon entropy, the fractal dimension is not sensitive to the molecular size. Collectively, the subgraphs encompass all features that are responsible for the complexity of the parent molecule, such as symmetry centers, branches, and rings. Therefore, all molecular features that were acknowledged to matter for molecular complexity are reflected by the fractal dimension, making it a holistic approach. And, the calculation of the fractal dimension for molecules works for all molecules. Every molecule contains substructures. And the number of unique substructures and their bond counts are the only parameters needed for the calculation.

Organic molecules cover a range of five fractal dimensions. The simplest organic molecules, linear alkanes have a fractal dimension of zero. They contain self-similar substructures, with identical number of details for all bond counts. Therefore, the number of bond counts versus the number of substructures is a straight horizontal line. Another class of molecules is represented by anandamide. The number of substructures reached a wide plateau at bond count five. Such a plateau will be observed for fatty acids and other molecules containing long chains. Glucose and strychnine represent complex organic molecules. The shape of the curve has a steep ascent until it reached the maximum. So, from the shape of the curve different classes of molecular fractality can be identified. Further investigation will show how the curve shape can be applied to support molecule classification in drug discovery. The calculation of fractal dimension provides a new matter constant for chemical structures and a new tool for evaluation of how accessible these structures are for synthesis. Molecular complexity has been a point of discussion for decades by organic chemists. The fractal dimension of molecules is a complexity measure that encompasses all molecular features, does not depend on the molecular weight, and straightforward to implement.

## Methods

The complete Java code for all calculations is available on GitHub https://github.com/Actelion/openchemlib. Details for the calculation of the fractal dimension are in the example folder examples/FractalDimension/README.md. A jar file with the compiled sources can be obtained from https://mvnrepository.com/artifact/com.actelion.research/openchemlib.
